# Efficacy of virtual reality therapy versus pharmacological sedation for reducing pain and anxiety during coronary catheterisation procedures: A prospective randomised controlled trial

**DOI:** 10.1002/hsr2.2151

**Published:** 2024-09-30

**Authors:** Julien Verain, Charlotte Trouillet, Fréderic Moulin, Charles Christophe

**Affiliations:** ^1^ Claude Bernard Clinical Hospital ELSAN Metz France

**Keywords:** anxiety, coronary angiography, hypnosis, pain, virtual reality

## Abstract

**Background and Aims:**

The use of virtual reality (VR) therapy has grown considerably, as it is effective for reducing pain and anxiety in different clinical areas. However, it has not been well evaluated for coronary angiography and angioplasty. This study aimed to compare VR therapy with pharmacological sedation (Sedation) for reducing pain in patients undergoing a planned coronary angiography or coronary/peripheral angioplasty.

**Methods:**

In this prospective randomized controlled trial, patients were randomly allocated to one of two groups before catheterization: a Sedation group (injection of midazolam and fentanyl) or a VR group (Deepsen VR headset). The primary outcome measure was the maximum pain during the procedure (visual analogue scale: 0–10). The secondary outcome measures were anxiety following the procedure (Spielberger State Anxiety Inventory: 20–80), the occurrence of arterial spasm, the haemodynamic profile and patient satisfaction.

**Results:**

The VR group (*n* = 63) had a mean pain rating of 2.5; for the Sedation group (*n* = 59) this was 1.0. This did not meet the criterion for non‐inferiority. Anxiety was comparable between the two groups (VR: 25.4; Sedation: 24.7), as was the occurrence of arterial spasm (VR: 7.9%; Sedation: 8.5%; *p* = 0.91), but blood pressure was higher in the VR group (140.2/71.7 mmHg vs. 121.8/64.7 mmHg). There were no VR‐related adverse effects, and patient satisfaction was high for both groups.

**Conclusions:**

Virtual reality therapy was not non‐inferior to pharmacological sedation for reducing pain during coronary angiography or angioplasty. However, it reduced anxiety to a comparable level. Virtual reality therapy represents an alternative to pharmacological sedation, which is well accepted by patients.

## 1 INTRODUCTION

In France, around 400,000 coronary angiographies and 200,000 coronary angioplasties are performed each year. As these endovascular procedures are minimally invasive, general anaesthesia is not required. A local anaesthetic (LoA) is administered at the site of the arterial puncture, usually the radial artery (91% of procedures in France), and conscious sedation can also be used.

Studies have shown that 24%–72% of patients experience clinically significant levels of anxiety before coronary angioplasty procedures. For example, one recent study on 100 patients found that it affected 49% of patients.^1^ Relatively few studies have assessed pain, but one study on patients undergoing a trans‐femoral angioplasty showed that the mean pain rating was 4.1 (Visual Analogue Scale; VAS: 0–10) when local anaesthesia alone was used, and 2.5 when patients were also given fentanyl.^2^

It is important to manage patients' pain and anxiety during these procedures, not only to ensure their comfort, but also to reduce the number of procedural difficulties and potential complications. It is also known that high levels of anxiety are linked to a poor recovery,^1^, ^3^ and perioperative pain relates to the incidence of postoperative complications.^4^ Furthermore, it has been shown that patient experience correlates with future adherence to treatment plans and other healthcare‐related behaviors (e.g., use of screening services).^5^

Sedatives are frequently used during coronary angioplasty procedures to reduce anxiety and pain, and they may also reduce the occurrence of arterial spasm.^6^ However, there can be side‐effects, including oxygen desaturation, a prolonged recovery time, confusion, amnesia and the need for antagonist drugs (flumazenil).^7^ Fortunately, these complications are usually benign, and they do not affect the patients' prognosis.^2^ However, although conscious sedation is the norm in many countries, particularly the United States,^2^ there are many medical centres throughout the world that cannot rely upon the availability of an anaesthetist.

An alternative possibility for managing patients' pain and anxiety involves the use of virtual reality (VR) therapy. This uses sight and sound to immerse patients in an artificial 3D environment created by a computer, which is designed to induce sedation and analgesia. The patients typically wear a headset that contains head‐movement sensors so that they can interact with the environment.^8^ There is evidence that this can be effective for reducing pain and anxiety during medical procedures.^9^ In addition, the side effects (e.g., nausea and vertigo) are mild and infrequent, affecting just 0%–8% of patients.^9^ However, at present, there is little data concerning the use of VR therapy during cardiac catheterization procedures. We are aware of one pilot study, which showed reduced anxiety during transcatheter aortic valve implantation,^10^ and another study, which showed less pain during atrial fibrillation ablation.^11^

In this study, we aimed to determine whether VR therapy is effective for reducing pain in patients undergoing a coronary angiography or angioplasty. For this, we compared a group of patients who received VR therapy during the procedure (without any sedative drugs) with a control group who received conscious sedation. We hypothesized that VR therapy is non‐inferior to moderate sedation for reducing pain during the procedure. We also examined secondary measures of interest: anxiety, the occurrence of arterial spasm, how well‐tolerated the device is, and the overall patient satisfaction.

## 2 MATERIALS & METHODS

### 2.1 Trial design

We carried out a monocentric, randomized, controlled, open‐label trial. The study was approved by the Committee for the Protection of Persons, registered on the site ClinicalTrials. gov (VirtuCardio: NCT05588232) and followed CONSORT guidelines.^12^

### 2.2 Participants

Patients were recruited from the private Claude Bernard Clinical Hospital in Metz, France. Screening was carried out by a research nurse on patients who were admitted for a planned coronary angiography, coronary angioplasty or peripheral angioplasty. The exclusion criteria were as follows: <18 years of age, refusal to participate in the study, a history of VR intolerance, epilepsy, severe cognitive impairment, language barrier, claustrophobia, nausea, under guardianship, pregnancy, and medical instability (shock, respiratory failure).

### 2.3 Interventions

Patients were allocated (1:1 ratio; simple randomization) to either the VR group or the Sedation group. This was carried out by a trained nurse using opaque sealed envelopes, as previously described.^13^ There was no blinding: the patient and the medical team were informed of the allocated group. An initial questionnaire was completed with the nurse to evaluate the patients' pain and anxiety before the procedure. The patients in the VR group were able to choose a VR session with the help of a brief description provided by the nurse. Patients were not administered any premedication.

In the catheterization laboratory, a nurse placed the VR headset (Deepson© VR system) on the patients in the VR group while the procedure was being prepared. The nurse checked that the device was working properly and stayed beside the patient throughout the procedure. The VR set‐up took around 2 min in total, and it was possible for the medical staff to pause the VR session, when necessary, to talk to the patient. The patients in the Sedation group were administered midazolam and fentanyl by slow intravenous injection during the set‐up. The dosage was determined by the patient's weight: <60 kg: 2 mg midazolam, 75 µg fentanyl; 60–100 kg: 3 mg midazolam, 75 µg fentanyl; >100 kg: 3 mg midazolam, 100 µg fentanyl. A supplementary bolus dose was administered every 45–60 min according to the patient's level of alertness and comfort, at the discretion of the anaesthetist and in line with recommendations.^5^ Patients in the Sedation group were continuously monitored by an anaesthetist or nurse anaesthetist throughout the procedure.

All patients were given LoA at the site of arterial puncture (subcutaneous lidocaine). For the trans‐radial and trans‐ulnar procedures, an injection of isosorbide dinitrate (1 mg) was administered to prevent spasm, and all patients were given unfractionated heparin (diagnostic procedures: 0.3 IU/kg; angioplasty procedures: 0.8 IU/kg). All patients in the Sedation group were given oxygen through a face mask; patients in the VR group were only given oxygen if the saturation was <94%.

Following the procedure, the patients went to the recovery room and a second questionnaire was completed with the nurse. The maximum amount of pain during the procedure was reported, and the level of anxiety following the procedure was assessed. Any vertigo or nausea during the procedure was noted. The level of immersion in the VR experience was also rated (VR group only) as well as the overall satisfaction with the procedure.

All serious adverse reactions were recorded from the time of inclusion up to discharge.

### 2.4 Outcomes

The primary outcome measure was the maximum pain experienced during the procedure. This was rated by patients on a VAS from 0 to 10 immediately following the procedure. The main secondary outcome measure was the level of situational anxiety, as assessed using the Spielberger State Anxiety Inventory (STAI‐Y).^14^ This anxiety scale assesses the anxiety response at a given time. We used a short, 6‐item version of the test in French, which has been validated for clinical research.^14^ Participants are asked to rate items on a scale from 1 to 4 (‘not at all’ to ‘very much so’), with total scores ranging from 6 to 24. These scores were multiplied by 3.34 (20/6) to give values from 20 to 80, as in the original version of the test, with the scores representing no anxiety up to very high levels of anxiety. This measure has been validated for patients with cardiovascular diseases, and a score >35 can be considered to represent clinically significant anxiety.^2^, ^15^, ^16^

Another secondary outcome measure was the occurrence of arterial spasm. This was rated by the operator at the end of the procedure, and was recorded as being absent, moderate or severe, based on the difficulty advancing the catheter. The operator also rated patient compliance during the procedure using three levels: no restlessness, moderate restlessness or major restlessness.

Other secondary outcome measures were obtained from the patients' post‐procedural questionnaire: the level of immersion in the VR experience, as rated on a VAS from 0 to 10, and the level of patient satisfaction. For the latter measure, patients were presented with statements and asked to respond on a Likert scale (disagree, somewhat disagree, somewhat agree, agree), for example “I am satisfied with the way that my pain and anxiety were managed during the procedure.” Patients in the VR group who had previously undergone a similar procedure were asked an additional question: “Did you prefer the procedure with a virtual reality headset?”

### 2.5 Statistical analysis

An analysis was run to estimate the number of patients needed to demonstrate the non‐inferiority of VR therapy compared with pharmacological sedation for reducing pain. This was run with the power set at 90%, a one‐sided significance level (α) of 2.5%, a non‐inferiority margin of 1, assuming that the actual difference between the two groups is 0, with a pooled standard deviation of 2 and with around 10% of patients whose data cannot be analysed. This analysis estimated that 200 patients would be needed (100 per group).

The full analysis set was analysed using SPSS version 27.0. The categorical variables were summarized using percentages, and quantitative variables were summarized using the mean and standard deviation. Percentages were compared using Chi‐square tests; means were compared using t‐tests, after checking the normality assumption. For the primary outcome measure (maximum pain during the procedure), the two groups were compared using an analysis of covariance (ANCOVA), which included the amount of pain before the procedure and the type of procedure (coronary angiography, coronary angioplasty or peripheral angioplasty). The 95% confidence intervals (CI) for the least square means were calculated for each group, and non‐inferiority was determined by a 95% CI upper limit less than 1 (clinically relevant non‐inferiority margin). The main secondary outcome measure of interest, the level of anxiety following the procedure, was expressed as a mean STAI‐Y score for each group. This was again analysed using ANCOVA, which included a measure of anxiety before the procedure and the type of procedure. Non‐inferiority was determined by a 95% CI upper limit less than five (clinically relevant non‐inferiority margin). The eta‐squared values for the ANCOVA adjustment variables (levels of pain and anxiety before the procedure, type of procedure) were recorded to show their effects. We used Cohen's interpretation of the values as follows: eta‐squared around 0.01: weak effect; around 0.06: moderate effect; around 0.14: strong effect.^17^

## 3 RESULTS

### 3.1 Study participants

**Figure 1:**
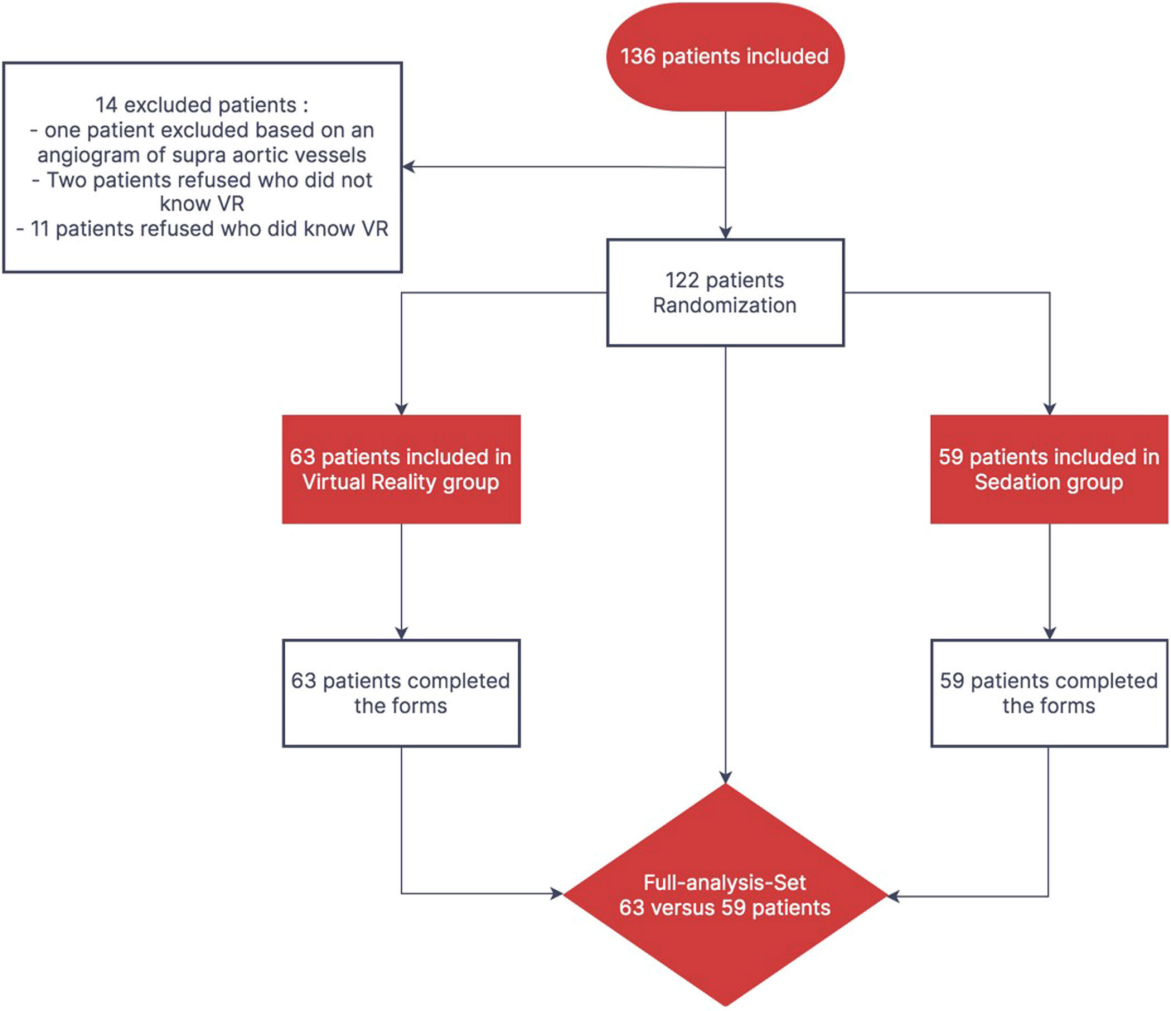
Flow chart of the study showing the patient inclusion, randomization into two groups and statistical analysis. VR, virtual reality.

Patients were included in the study between October 2021 and February 2022. The study was ended due to a lack of staff, which resulted from the COVID‐19 pandemic. For the full analysis set, there were 122 patients (Figure 1). The mean age was 68.7 ± 9.8 years, 75.4% were men, 56.6% had previously had a coronary angiography, 41.0% had a history of coronary artery disease or vascular disease, and 9% had a history of anxiety or depressive disorders with long‐term treatment using psychotropic drugs. The patients in the VR and Sedation groups did not differ significantly in terms of their baseline clinical characteristics (Table 1).

**Table 1:** Characteristics of the patients and procedures, with statistical comparison of the virtual reality and sedation groups.

	Total FAS (N = 122)	Virtual reality group (N = 63)	Sedation group (N = 59)	*p*
Patient characteristics				
Patients, N (%)	122	63 (51.6)	59 (48.4)	‐
Sex: male, N (%)	92 (75.4)	50 (79.4)	42 (71.2)	0.29^b^
Age, years (mean ± SD)	68.7 ± 9.8	68.5 ± 10.0	68.8 ± 9.5	0.84
BMI, kg/m^2^ (mean ± SD)	28.6 ± 5.2	28.2 ± 5.4	29.0 ± 5.0	0.37
Previous coronary angiography, N (%)	69 (56.6)	36 (57.1)	33 (55.9)	0.89^b^
History of vascular disease or ischaemic heart disease, N (%)	50 (41.0)	27 (42.9)	23 (39.0)	0.66^b^
Diabetes, N (%)	42 (34.4)	22 (34.9)	20 (33.9)	0.91^b^
Arterial hypertension, N (%)	81 (66.4)	38 (60.3)	43 (72.9)	0.14^b^
Chronic anxiety or depressive disorders, N (%)	11 (9.0)	6 (9.5)	5 (8.5)	0.84^b^
Procedures				
Coronary angiography/arteriography, N (%)	70 (57.4)	39 (61.9)	31 (52.5)	0.33^b^
Coronary angioplasty, N (%)	46 (37.7)	20 (31.7)	26 (44.1)	0.33^b^
Peripheral angioplasty, N (%)	4 (4.9)	4 (6.3)	2 (3.4)	0.33^b^
Stable coronary artery disease, N (%)	103 (84.4)	51 (81.0)	52 (88.1)	0.37^b^
Acute coronary syndrome, N (%)	7 (5.7)	4 (6.3)	3 (5.1)	0.37^b^
Stable peripheral artery disease, N (%)	9 (7.4)	5 (7.9)	4 (6.8)	0.37^b^
Critical peripheral artery disease, N (%)	3 (2.5)	3 (4.8)	0 (0)	0.37^b^
Procedure duration, minutes (mean ± SD)	27.2 ± 18.5	26.4 ± 18.7	28.2 ± 18.4	0.59
Total dose area product, Gy cm^2^ (mean ± SD)	4922 ± 5203	4691 ± 4829	5169 ± 5607	0.62
Amount of iodinated contrast, ml (mean ± SD)	114 ± 59.9	110 ± 60.2	118 ± 59.9	0.50
Trans‐radial approach, N (%)	108 (88.5)	58 (92.1)	50 (84.7)	0.30^b^
Trans‐ulnar approach, N (%)	5 (4.1)	1 (1.6)	4 (6.8)	0.30^b^
Trans‐femoral approach, N (%)	9 (7.4)	4 (6.3)	5 (8.5)	0.30^b^
Diametre of the introducer, Fr (mean ± SD)	5.5 ± 0.5	5.4 ± 0.5	5.6 ± 0.6	0.04

Abbreviations: BMI, Body mass index; FAS, Full analysis set; SD, standard deviation.

^a^ Chi^2^ test

^b^ T‐test

In this study, 57.4% of the patients underwent a diagnostic procedure (coronary angiography), 37.7% a coronary angioplasty, and 4.9% a peripheral angioplasty. The mean duration was 27.2 min. There were 103 patients (84.4%) with stable coronary artery disease, and just seven (5.7%) with acute coronary syndrome (ACS); this can be attributed to the study being restricted to planned procedures. The two groups did not differ significantly in terms of the procedural characteristics, apart from the introducer diameter, which is not clinically relevant.

### 3.2 Pain during the procedure

There was no pain (VAS = 0) before the procedure for 86% of the patients in the VR group and 85% in the Sedation group. After adjusting for pain before the procedure and the type of procedure, the maximum pain during the procedure was rated to be 2.5 on average for the VR group and 1.0 for the control group. The difference between the two groups was 1.4, which does not meet the criterion for non‐inferiority (Table 2).

**Table 2:** Maximum pain during the procedure.

Maximum pain during the procedure (VAS)			
	**Virtual reality group (*N*** = **61)**	**Sedation group (*N*** = **51)**	*p*
Adjusted mean ± SE (CI)	**2.5** ± 0.28 (1.9 – 3.0)	**1.0** ± 0.30 (0.4 – 1.6)	**<0.001**
Difference between groups, mean ± SE (CI)	**1.4** ± 0.41 (0.6 – 2.2)		

*Note*: Bold values are statistically significant.

Abbreviations: CI, 95% confidence intervals; SE, standard error; VAS, visual analogue scale.

**Figure 2:**
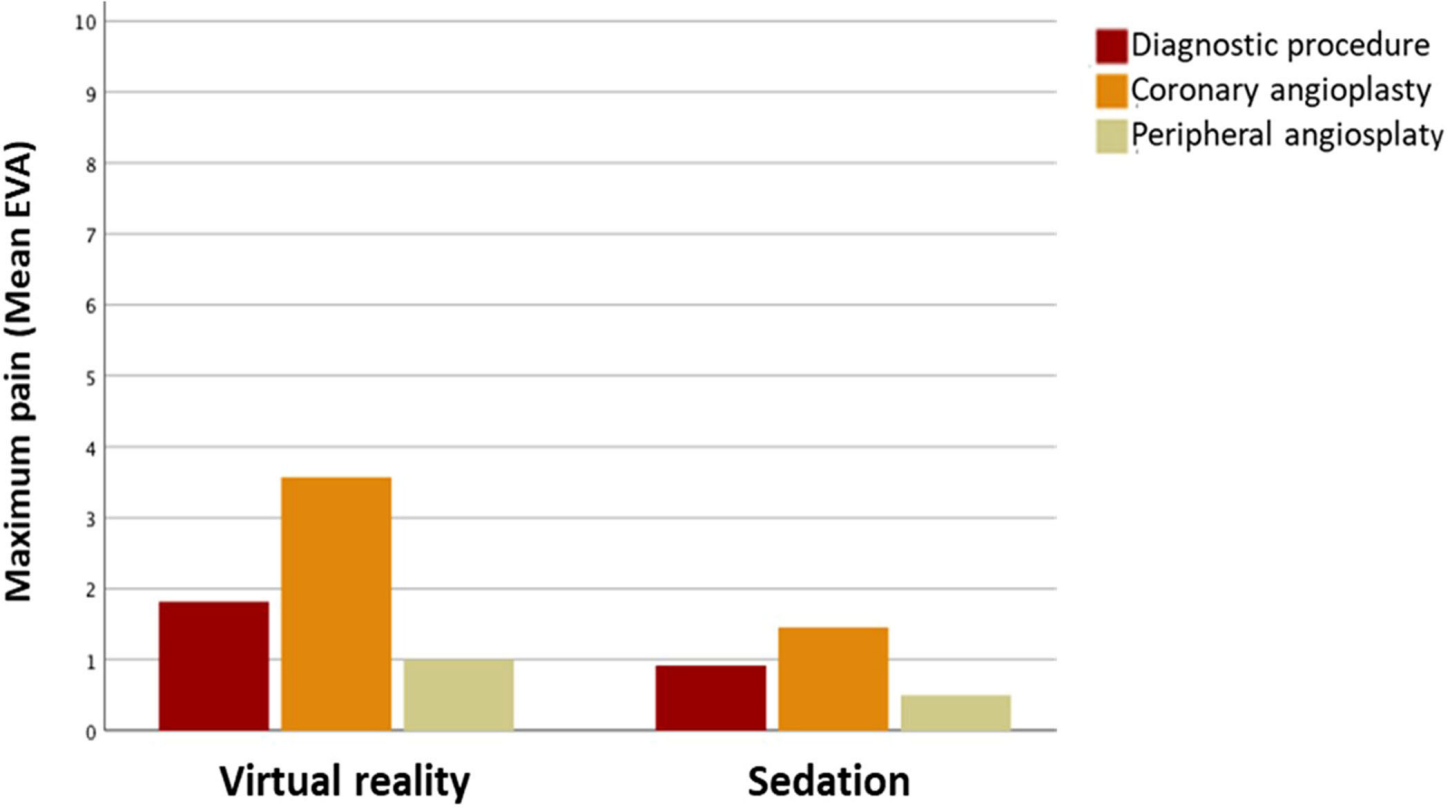
Maximum pain according to the type of procedure. VAS, Visual Analogue Scale.

The maximum pain for the different procedures is shown in Figure 2. The type of procedure had little effect on the pain, irrespective of the group (eta‐squared 0.008).

For the two groups combined, 84% of patients reported the most painful area to be the site of arterial puncture. Only one patient required an additional dose of analgesia during the procedure (Sedation group patient; pain rating: 10/10).

### 3.3 Peri‐procedural anxiety

After adjusting for the preprocedural anxiety (VR group: 30.5 ± 11.3; Sedation group: 31.4 ± 13.7) and the type of procedure, the STAI‐Y score following the procedure was 25.4 for the VR group and 24.7 for the Sedation group (Table 3). The difference between the groups was 0.75, which meets the criterion for the non‐inferiority of VR and is not statistically significant (*p* = 0.66). The preprocedural anxiety had a moderate effect on the post‐procedural anxiety (eta‐squared: 0.08), and the type of procedure had a weak effect (eta‐squared: 0.01). The preprocedural anxiety was not found to correlate with the pain during the procedure (Pearson's *r* = 0.18, *p* = 0.08).

**Table 3:** Anxiety following the procedure.

Anxiety following the procedure (STAI‐Y score)			
	Virtual reality group (*N* = 49)	Sedation group (*N* = 44)	*p*
Adjusted mean ± SE (CI)	**25.4** ± 1.17 (23.1 – 27.7)	**24.7** ± 1.22 (22.3 – 27.1)	0.66
Difference between groups, mean ± SE (CI)	**0.75** ± 1.69 (−2.6 – 4.1)		

*Note*: Bold values are statistically significant.

Abbreviations: CI, 95% confidence intervals; SE, standard error; STAI‐Y, Spielberger State Anxiety Inventory.

### 3.4 Safety and tolerability

There were no reports of nausea, emesis or vertigo, and there were no cases of postinterventional delirium. Arterial spasm was reported for five patients (7.9%) in the VR group and five (8.5%) in the Sedation group (*p* = 0.91); these cases did not necessitate a change in the approach. In the VR group, 4.8% (3/63) of patients were reported to be restless, compared with 11.9% (7/59) in the Sedation group (*p* = 0.15).

Compared with the Sedation group, the VR group had higher systolic blood pressure (140.2 mmHg vs. 121.8 mmHg; *p* < <  0.001) and higher diastolic blood pressure (71.1 mmHg vs. 64.7 mmHg; *p* < 0.001; Table 4). Seven patients in the VR group were given antihypertensive medication (nicardipine) during the procedure, compared with just one patient in the Sedation group. Three patients had grade 3 hypertension (systolic blood pressure >180 mmHg), all in the VR group. The mean heart rate did not differ significantly between the two groups (*p* = 0.87).

**Table 4:** Haemodynamic profile.

Haemodynamic Profile			
	Virtual reality group (*N* = 63)	Sedation group (*N* = 59)	*p*
Systolic blood pressure, mmHg; mean ± standard deviation	140.2 ± 22.6	121.8 ± 19.4	<0.001
Diastolic blood pressure, mmHg; mean ± standard deviation	71.1 ± 11.0	64.7 ± 9.1	<0.001
Antihypertensive medication administered, n (%)	7 (11.1%)	1 (1.7%)	NS
Heart rate/min, mean ± standard deviation	71.7 ± 12.7	71.3 ± 13.3	0.87

### 3.5 Feasibility of virtual reality therapy

The VR headset was worn by 92.1% of the VR group patients throughout the whole procedure. Of those who did not, two had poor visual adaptation to the VR, one experienced pain at the back of the head, and the battery ran out for two patients. The VR therapy was found to be well‐tolerated; this is noteworthy as 30.2% of the patients were older than 74.

The degree of immersion in the VR experience was rated as 6.2/10 on average. This rating correlated negatively with the post‐procedural anxiety (Pearson's *r* = −0.30; *p* = 0.03).

### 3.6 Patient satisfaction

The great majority of patients were satisfied with the way that their pain and anxiety were managed during the procedure (VR group: 96.7%; Sedation group: 100%; Table 5). If they were to undergo the procedure again, 88.3% of patients in the VR group reported that they would want to have VR therapy. Of the 41 patients in the VR group who had previously undergone cardiac catheterization without VR therapy (LoA alone or sedation), 87.7% preferred the procedure with VR therapy.

**Table 5:** Patient satisfaction.

Patient satisfaction questionnaire	Virtual reality group (*N* = 61)	Sedation group (*N* = 57)
Responded “agree” or “somewhat agree”	%	%
“I am satisfied overall with the care I have received during my time at hospital”	98.4	98.1
“I am satisfied with the way that my pain and anxiety were managed during the procedure”	96.7	100
“If the procedure had to be repeated, I would like the same form of pain and anxiety relief using a virtual reality headset ‐ or pharmacological sedation”	88.3	91.2

## 4 DISCUSSION

To the best of our knowledge, this is the first randomized controlled trial to assess the use of VR therapy versus pharmacological sedation for patients undergoing coronary angiography or angioplasty. The results showed that VR therapy was not non‐inferior to sedation for reducing pain during the procedure. However, anxiety was reduced to comparable levels.

In this study, the patients generally reported mild levels of pain (VAS < 3). Although few previous studies have analysed pain during coronary catheterization procedures, it is known that the introduction of the trans‐radial approach led to a reduction in the amount of pain and discomfort. This approach was used for 88.5% of our patients. For the trans‐femoral approach (used for 7.4% of our patients), there is typically pain and discomfort when the introducer is removed.^1^, ^18^ It could therefore be argued that the use of powerful analgesics, such as fentanyl, is appropriate for this approach, but not for others that are associated with less pain. There are clear advantages in avoiding sedative drugs in terms of the costs, supply issues, the need for anaesthetists, and the risk of side effects. Although serious adverse events resulting from sedation are rare,^5^ we observe minor side effects almost daily. For instance, drowsiness, confusion and anterograde amnesia regularly lead to the cancellation of a same‐day discharge and sometimes lead to further tests, such as brain scans.

Our study found that there were fewer restless patients in the VR group than the Sedation group, although this was not statistically significant. By focusing their attention on the VR environment, which is designed to foster relaxation, patients can remain calm and tolerate staying still for long periods. Although this can also be achieved using pharmacological sedation, there is a risk of paradoxical agitation when using these drugs. As the level of immersion in the VR environment was not optimal in this study (6.2/10), there is scope for further improvement.

Previous studies have shown than patients experience anxiety before an angioplasty due to the fear of procedure‐related complications, disease progression and pain during the procedure.^2^ The anxiety can manifest itself as fear, tension and feelings of panic, which are associated with the occurrence of chest pains and cardiac complications.^6^ There is also evidence that anxiety is closely connected to pain.^19^ In our study, we found moderate levels of anxiety before the procedure, with 34% of patients having clinically significant anxiety (STAI‐Y ≥ 35). This is lower than in a previous study, where 49% of patients were affected.^2^ This could be attributed to technical developments over the last 12 years, such as the use of the trans‐radial approach and outpatient procedures.

Previous research using similar sedatives has shown that moderate sedation reduces the incidence of arterial spasm in patients undergoing trans‐radial coronary angioplasty.^3^ In our study, the occurrence of arterial spasm did not differ significantly between the VR and Sedation groups, and there were no cases of severe spasm. This is an encouraging result, but larger studies are needed to confirm this non‐inferiority.

Our findings indicate that VR therapy is effective for reducing patients' anxiety during coronary catheterization procedures. This result may also apply to other catheterization procedures, such as cerebral angiography, as well as to younger patient groups. However, our study has several limitations.

The main limitation is that there were fewer patients than initially planned. According to our power analysis, there should have been 100 patients per group. This was not achieved because the study was discontinued due to a lack of staff. A second limitation relates to the open‐label design and the resulting potential for bias. For example, in the absence of sedation, operators may have administered LoA more effectively and taken greater care to avoid catheter movements that may be painful. This would have affected the pain ratings. A third methodological limitation relates to the primary outcome measure, the pain VAS, which is subjective and has low reproducibility. As an alternative, “objective” measures could be used that correlate with pain, such as blood pressure and the heart rate. However, in our study, the higher blood pressure in the VR group could not be attributed to pain, as sedation is known to lower blood pressure.^5^ In addition, the heart rate was found to be similar for the two groups. Of note, our secondary outcome measure, anxiety, is also subjective; however, the STAI‐Y has been well‐validated in both experimental and clinical settings. A final limitation is that arterial spasm was clinically evaluated by the operator and not confirmed angiographically; however, this was only a secondary exploratory measure.

In the future, studies could explore the use of VR therapy alongside top‐up doses of analgesics during coronary catheterization procedures. It would also be of interest to determine the benefit of running VR sessions before the procedure when anxiety is at its peak. Further work is required to identify the mechanisms by which VR therapy affects patients, and to maximize the VR content, focusing on the most effective elements.

## 5 CONCLUSIONS

Although VR therapy was not found to be non‐inferior to pharmacological sedation for reducing pain during coronary angiography or angioplasty procedures, it reduced anxiety to comparable levels. These findings indicate that VR therapy could be effective for reducing patients' anxiety, but not their pain, during these procedures.

### 5.1 Impact on daily practice

The results of this study indicate that virtual reality therapy could be used to reduce anxiety during coronary catheterization procedures. Although it does not appear to reduce the pain felt by patients, it could be of use for procedures with lower levels of pain, such as those with a trans‐radial approach. Virtual reality therapy was found to be well‐accepted by the older adults in this study, thus supporting its potential usefulness for these procedures.

## AUTHOR CONTRIBUTIONS

**Julien Verain**: Conceptualization; formal analysis; investigation; writing—original draft. **Fréderic Moulin**: Investigation. **Charlotte Trouillet**: Investigation. **Charles Christophe**: Conceptualization; investigation.

## CONFLICT OF INTEREST STATEMENT

The authors have no conflicts of interest to declare.

## ETHICS APPROVAL STATEMENT

The study was approved by the Committee for the Protection of Persons in Poitiers, France (CPP Ouest III) on October 15, 2021.

## PATIENT CONSENT STATEMENT

All study participants, or their legal guardian, provided informed written consent before study enrolment.

## CLINICAL TRIAL REGISTRATION

This study was registered on the site ClinicalTrials. gov (VirtuCardio: NCT05588232).

## TRANSPARENCY STATEMENT

The lead author Julien Verain affirms that this manuscript is an honest, accurate, and transparent account of the study being reported; that no important aspects of the study have been omitted; and that any discrepancies from the study as planned (and, if relevant, registered) have been explained.

## Data Availability

The data that support this study's findings are available from the corresponding author (JV) upon reasonable request.
